# The Effect of Probiotics on the Prognostication of the Neutrophil-to-Lymphocyte Ratio in Severe Multi-Trauma Patients

**DOI:** 10.3390/jpm14040419

**Published:** 2024-04-15

**Authors:** Alexandra-Eleftheria Menni, Georgios Tzikos, Barbara Fyntanidou, Aristeidis Ioannidis, Lamprini Loukipoudi, Vasilis Grosomanidis, Angeliki Chorti, Anne Shrewsbury, George Stavrou, Katerina Kotzampassi

**Affiliations:** 1 Department of Surgery, Aristotle University of Thessaloniki, 54636 Thessaloniki, Greecegtziko@auth.gr (G.T.); ariioann@yahoo.gr (A.I.); chorange@auth.gr (A.C.); a_shrewsbury@yahoo.com (A.S.); 2 Department of Emergency Medicine, Aristotle University of Thessaloniki, 54636 Thessaloniki, Greece; fyntanidou@auth.gr; 3 Department of Anesthesia & Intensive Care, Aristotle University of Thessaloniki, 54636 Thessaloniki, Greece; linaloukipoudi@gmail.com (L.L.); vgrosoma@auth.gr (V.G.); 4 Department of General Surgery, Addenbrooke’s Hospital, Hills Road, Cambridge CB2 2QQ, UK; stavgd@gmail.com

**Keywords:** neutrophil-to-lymphocyte ratio (NLR), probiotics, multi-trauma, traumatic brain injury, ventilator-associated pneumonia, prognostic factor, lymphocytopenia, immunomodulation

## Abstract

Background: The ratio of neutrophils to lymphocytes [NLR] is one of the most accepted prognostic indices and demonstrates a positive correlation with the severity of a disease. Given that probiotics exerted immunomodulatory properties and thus positively affected lymphocytopenia induction in severely ill patients, we performed a post hoc analysis in the ProVAP protocol to investigate whether probiotics affected the prognostication of NLR in respect to ventilator-associated pneumonia in multi-trauma patients. This cohort mandatorily involved severe traumatic brain injury patients. Methods: The white blood cell data of all patients, after being retrieved for the days 0 and 7, were statistically assessed in respect to neutrophils, lymphocytes and NLR among the 4 sub-groups of the study: placebo/no-VAP, placebo/VAP, probiotics/no-VAP, and probiotics/VAP. Results: Lymphopenia was dominant in placebo sub-groups, while an increased level of lymphocytes was prominent in probiotics sub-groups. This resulted in an increase [*p* = 0.018] in the NLR value in the probiotics/VAP group in relation to the probiotics/no-VAP cohort; this was an increase of half the value of the placebo/VAP [*p* < 0.001], while the NLR value in placebo/no-VAP group increased almost four-fold in relation to probiotics/no-VAP [*p* < 0.001]. Additionally, the ROC curve for probiotic-treated patients revealed a NLR7 cut-off value of 7.20 as a prognostic factor of VAP (AUC: 78.6%, *p* = 0.015, 95% CI: 62.6–94.5%), having a high specificity of 90.2% and a sensitivity of 42.9%. Conclusions: NLR may considered a credible prognostic biomarker in multi-trauma patients since it can evaluate the immunomodulatory benefits of probiotic treatment. However, the results of the present post hoc analysis should be interpreted meticulously until further evaluation, since they may be basically species- or strain-specific.

## 1. Introduction

Severe trauma, especially when brain injury is involved, is recognized as one of the main reasons for complications and mortality. Multiple-trauma patients either die as a direct consequence of their injuries, or, more commonly, days later, after the development of secondary infectious complications [1,2,3,4,5]. Despite the progress in ICU therapeutic measures, the incidence of mortality remains practically unaffected: in 2004, Osborn et al. reported a 23% mortality rate [6], and almost 20 years later, in 2021, Sy et al. presented a similar rate of 24.3% [7].

Today, the gut continues to be “the motor” of critical illness, although issues present in a more sophisticated manner: severe trauma, stressful surgical interventions, or sepsis, for example, trigger the disruption of the homeostasis of the microbiota, leading within hours to the loss of bacterial heterogeneity, a reduction in the number of commensal phyla, and thus the prevalence of pathogens. This further triggers and promotes harm to the already-compromised host in a positive feedback spiral [8].

Following severe injury and the consequent systemic inflammatory response, the host’s archetypal response is an increase in neutrophils and a decrease in lymphocytes, with it being well accepted that the neutrophil is the predominant cellular component of host defense against infectious injury. At the same time, the lymphocytes are known as the predominant cellular components of the adaptive immune system [9]. Zahorec [10] has noted a connection between disease severity and the presence of lymphopenia and neutrophilia, since the failure of the host to revert lymphopenia to a normal state has been linked to increased mortality, regardless of the type of leukocytosis [11].

The complete blood count test is one of the cheapest and easiest analyses to implement in a clinical laboratory examination and provides useful information on all blood cell types, and more particularly on neutrophils and lymphocytes—the most implicated in the progression of many diseases. Thus, the ratio of neutrophils to lymphocytes (NLR) remains a reliable inflammatory indicator of a high prognostication. Additionally, it is easy to calculate, and can be determined at almost zero cost [9,10,12].

Probiotics, defined by the FAO [Food & Agricultural Organization] and the WHO [World Health Organization] as “live microorganisms, which, when consumed in adequate quantities, confer a health benefit on the host” [13], were introduced early into the armamentarium used against critical illness to stimulate and augment the immunological response of the host, to produce antimicrobial compounds, and to competitively eliminate pathogens [14,15,16,17]. Recently, it became evident that they are able to promote the healing process after tissue injury, both through the restoration of the compromised local and systemic microbiome and the enhancement of anti-inflammatory, healing, and angiogenetic factors [18,19,20].

Given that the “upon intubation” initiation of probiotic treatment in mechanically ventilated multi-trauma individuals—including cases of brain injury—was found to significantly decrease the prevalence of severe infectious complications [14,21,22], we decided to test the applicability of the NLR as a prognostic index of ventilator-associated pneumonia [VAP] induction in such patients, allocated to probiotic treatment or placebo groups. We aimed to assess whether there was a difference in the NLR critical value [cut-off point] between placebo and probiotic-treated patients in the prognostication of VAP infection, and to interpret the difference, if any, through the immunomodulatory properties of the probiotics.

## 2. Material and Method

### 2.1. Study Design

Data that had been prospectively gathered for the ProVAP trial [22], registered in Clinicaltrials.gov as NCT03074552, were retrospectively evaluated. ProVAP was a randomized, double-blind trial held in seven surgical ICUs in Thessaloniki and Northern Greece. This study was performed on severely injured patients, who had been immediately intubated, either in the pre-hospital setting or by the emergency services upon arrival. Severe traumatic brain injury and at least one more organ-system injury were the mandatory criteria for the inclusion of patients in the study, the primary aim of which was the investigation of the efficacy of a multi-probiotic regime for the prevention of VAP. Additional criteria for a patient to be eligible for participation were being an adult aged 18 to 80 years; being intubated and starting mechanical ventilation immediately after injury; remaining under mechanical ventilation for more than 10 days; and the life expectancy being more than 15 days. Exclusion criteria were pregnancy or lactation; immunosuppression due to any reason; vascular graft surgery in the past or a prosthetic heart valve; a recent cardiac or major artery trauma; oral or pharyngeal trauma; and a body mass index of more than 40 kg/m^2^. Moreover, recent infections, especially of the respiratory tract or sinusitis, as well antibiotic and/or probiotic treatment for more than 3 days of screening were grounds for exclusion [22].

A legal proxy signed an informed consent for study participation. Eligible participants were administered a probiotic combination or placebo, as assigned by a computer-generated table. The allocation was blinded to both investigators and the physicians of the yard. The preparation being tested [LactoLevure^®^, Uni-Pharma, Athens, Greece] was a combo formula of four probiotics per container: *L. acidophilus* LA-5 1.75 × 10^9^ cfu, *L. plantarum* 0.5 × 10^9^ cfu, *B. lactis* BB-12 1.75 × 10^9^ cfu, and *S. boulardii* 1.5 × 10^9^ cfu, while the control containers [placebo]—being the same in appearance—enclosed maltodextrine in powder form. Every participant received two containers, twice per day, for two weeks; the first, diluted into 100 mL of tap water, was administered via a Levin catheter or via a PEG, and the other was a gel applied to the oropharynx, as described in the previous report [22].

Ventilator-associated pneumonia is well defined as any infection of the lower respiratory tract presenting all of the following criteria: onset > 48 h after mechanical ventilation initiation; increase in SOFA score by more than 1 point; presence of a new infiltrate in chest X-ray or CT; increase in body temperature over 38 °C; tracheal–bronchial secretions of a purulent nature; clinical pulmonary infection score over 6 [23]; and isolation of a pathogen from BAL fluid through a quantitative culture or through molecular detection by counting the number of bacteria [concentration higher than 1 × 10^4^ cfu/mL].

### 2.2. Data Collection

For the present study, the data relating to the white blood cell counts were gathered from the patients’ files on days 0 and 7. The decision of which day constituted day 7 was based on the day the first insult of ventilation-associated pneumonia occurred. From these data, only the neutrophil and lymphocyte values [in absolute numbers and not as a percentage] were used. The absolute number of neutrophils was then divided by the absolute number of lymphocytes on a given day, the result being the NLR value.

After the initial statistical evaluation of these parameters [neutrophils, lymphocytes and NLR] per study group [probiotics or placebo], all parameters were re-assessed after the two groups were sub-divided into those with uneventful postoperative courses and those who experienced VAPs.

### 2.3. Statistical Analysis

The normality of data distribution was estimated using the Kolmogorov–Smirnov test, since both groups included more than 50 participants. For continuous variables, the results were expressed as mean ± SD. In order to evaluate the statistical difference between values at two time points (day 0 and day 7) within the same group the paired *t*-test was applied, while the *t*-test for independent variables was used for comparisons of the same values between groups [placebo vs. probiotics and no-VAP vs. VAP].

Additionally, an ROC curve was generated in order to calculate the optimal cut-off point of NLR for predicting VAP. The cut-off point was selected based on the accompanying Youden’s index, while sensitivity and specificity rates were also calculated [24,25]. Statistical analysis was performed using the Statistical Package for Social Science (SPSS), Inc. (v 25.0; Chicago, IL, USA) was used for the statistical analysis, with the level of statistical significance set at 0.05.

## 3. Results

Data from one hundred and twelve participants were analyzed; fifty-three patients received the placebo treatment and fifty-nine received the probiotic regime, as already presented in the initial publication. Their baseline characteristics regarding age and gender, as well as Acute Physiology and Chronic Health Evaluation (APACHE) II score; Simplified Acute Physiology Score II (SAPS); Sequential Organ Failure Assessment (SOFA) score; Charlson’s Comorbidity Index (CCI)score; Simplified Acute Physiology Score (SAPS); and GCS scores were all comparable in placebo and probiotic-treated patients, as was presented in the previous report [22].

### 3.1. Neutrophils

On day 0, upon admission, the neutrophils were found to be within normal values, both in the placebo and probiotics groups, while on day 7—as expected—neutrophil values were significantly increased to almost double in both probiotic- and placebo-treated groups [*p* < 0.001—Table 1].

However, when patients from each group were sub-divided into those presenting with VAP or not during the course of their hospitalization, there were significant differences within the placebo and probiotic groups: placebo-treated patients who did not experience VAP [no-VAP group] were found to have a statistically highly significant increase in neutrophils [*p* < 0.001] between days 0 and 7; and the same was observed in the probiotics/no-VAP group. However, the placebo/VAP group showed a smaller increase between days 0 and 7 [*p* = 0.012] compared to the probiotics/VAP group for the same days [*p* = 0.001—Table 2].

### 3.2. Lymphocytes

On day 0, the lymphocytes were found to be within normal values, both in the placebo and probiotic groups. However, on day 7, lymphocyte counts were significantly decreased to almost half the initial values in placebo patients [from 1.30 ± 0.37 to 0.86 ± 0.43, *p* < 0.001], whereas they were significantly increased [from 1.23 ± 0.29 to 2.95 ± 1.23, *p* < 0.001] in probiotic-treated patients, in relation to day 0 [Table 3].

When patients from each group were sub-divided into those who presented with no-VAP or with VAP during the course of their hospitalization, significant differences were prominent between placebo and probiotic patients. Both sub-groups of placebo-treated patients were found to have a reduction in lymphocyte count on day 7. Placebo/no-VAP had a statistically significant drop [*p* < 0.001], while placebo/VAT showed an insignificant reduction. However, both probiotic-treated sub-groups exhibited significant increases, which were more pronounced in the probiotic/no-VAP group [*p* < 0.001—Table 4].

### 3.3. Neutrophil-to-Lymphocyte Ratio

The calculated NLR was to be found similar in both the placebo and probiotic groups on admission [day 0]. However, on day 7, the NLR was highly significantly increased [up three times, *p* < 0.001] in the placebo, while remaining almost stable in the probiotic-treated patients, which was probably due to the increased number of lymphocytes [involved in the NLR ratio as denominator] present in this group [Table 5].

When each group was sub-divided into those who presented VAP or not during their hospitalization, no significant difference was found for the placebo group; the NLR value on day 7 was triple the initial value in both sub-groups. However, probiotic administration in the no-VAP group led to a decrease in the NLR value [*p* = 0.01], while in probiotic/VAP patients the NLR was found to be increased [*p* = 0.018]. However, this increase was—numerically—half the value of that of the corresponding placebo/VAP sub-group [15.45 *±* 5.19 vs. 8.8 *±* 1.11, *p* < 0.001], while the NLR value in the placebo/no-VAP was increased almost four-fold in relation to the probiotics/no-VAP sub-group [16.43 *±* 6.07 vs. 4.18 *±* 2.22, *p* < 0.001] [Table 6].

### 3.4. ROC Curve and Optimal Cut-Off Points

**Figure 1:**
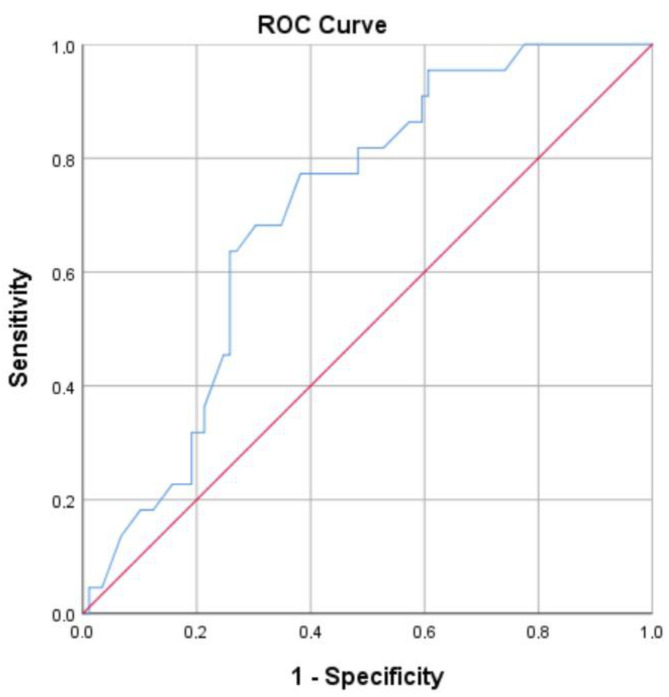
ROC curve analysis of all patients’ NLR7 data.

After the generation of the ROC curve (Figure 1) by inserting all the patients’ NLR7 data, we found the NLR7 value to have a good discriminatory performance for the prediction of VAP, with an optimal cut-off point [based on Youden’s index] equal to 10.55 (AUC: 70.7%, *p* = 0.003, 95% CI: 60.2–81.2%), giving a sensitivity of 77.3% and a specificity of 61.8%. In addition, after further evaluation to achieve greater sensitivity, equal to 81.8%, but reduced specificity [51.7%], the cut-off value was found to be 7.20.

**Figure 2:**
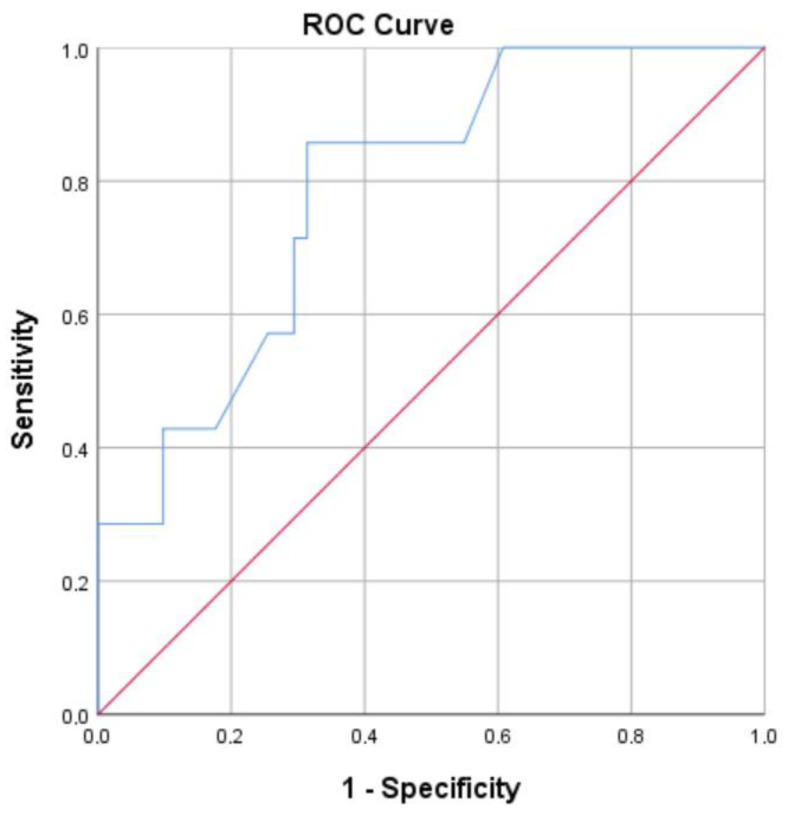
ROC curve analysis of probiotic-treated patients’ NLR7 data.

Following this, we evaluated the NLR7 predictive value of placebo and probiotic groups. No statistically significant predictive value was found for NLR7 (AUC: 53.2%, *p* = 0.715, 95% CI: 37.1–69.4%) in the placebo group. On the contrary, the ROC curve (Figure 2), generated by analyzing probiotic-treated patients’ NLR7 data, revealed the NLR7 to be a reliable predictive factor for VAP (AUC: 78.6%, *p* = 0.015, 95% CI: 62.6–94.5%). Furthermore, looking for the “best specificity” cut-off point, we determined it to be 7.20, with a specificity of 90.2% and a sensitivity of 42.9%. In other words, in patients treated with probiotics, a NLR7 value equal or higher than 7.20 may be considered an alert for a VAP induction.

## 4. Discussion

In this analysis, we tried to investigate the applicability of the NLR, calculated on the 7th post-trauma day, as a prognostic index of VAP infection in patients that were treated with either a placebo or a four-probiotic regime. Given that [i] the ProVAP multi-center study [22] revealed that immediate probiotic administration significantly reduced the frequency of VAP, and becaise [ii] probiotics are well documented to exert immunomodulatory properties [26,27], we anticipated that the NLR, as the proportion of neutrophils to lymphocytes, would be reduced and may thus thought to be in doubt as a prognostic index of VAP manifestation.

The two groups of patients included in the ProVAP study on which the post hoc analysis is based exhibited an outstanding homogeneity in their characteristics regarding age—being under 55 years old—gender and APACHE II, SAPS II, SOFA, CCI, and GCS scores. They also had the same comorbidities and received the same antibiotics and enteral nutritional support [22]. All participants had experienced multi-traumas, which were required to involve severe brain trauma and—at minimum—one more organ system, and thus the necessity of immediate intubation either in the pre-hospital setting or the emergency department upon arrival [28]. The severity of trauma, as a consequence, was required to induce prolonged intubation and mechanical ventilation—at least for the next 10 days—in order to have put the patients at a significant risk of developing VAP.

Brain trauma in general is well known to represent a complex biochemical cascade related with numerous patho-physiological functions, affecting not only the central nervous system but the function of multiple distant organs/systems [29,30,31]. Furthermore, acute brain trauma was found to significantly deteriorate gut microbiota diversity—starting within hours of injury [32]. Feces, recovered by means of rectal swabs from 101 patients suffering moderate-to-severe traumatic brain injuries, were found to be extensively colonized by *Proteobacteria* phyla, with the *Enterobacteriaceae* being the predominant group [33]. Other authors reported a reduction in *Bacteroidetes*, *Firmicutes, Verrucomicrobia* and *Faecalibacterium* and an increase in pathogens, such as *Enterococcus*, *Parabacteroides*, and *Lachnoclostridium*, associated with chronic inflammatory conditions [34,35]. Furthermore, brain trauma is linked with gut barrier dysfunction, previously known as “leaky” gut [36,37]. Alterations in microbiota diversity are recognized as contributing to infections, and mainly to pneumonia, through the gut–brain–lung axis [38,39,40,41,42], while the disruption of the intestinal barrier, as the result of pathogen overgrowth and loss of the beneficial role of the commensal bacteria, contributes to long-term complications [43,44,45,46] due to the deterioration of host immunity [47].

In the present analysis, the number of probiotic-treated patients presented VAP during their ICU stay was significantly small [*n* = 7 out of 59 patients, rate 11.9%] in relation to the placebo-treated group [*n* = 15 out of 53 patients, rate 28.3%, *p* = 0.034]. This post hoc analysis showed a significant reduction in the NLR index in this patient group, compared to the corresponding group of placebo/VAT patients [8.8 ± 1.11 vs. 15.45 ± 5.19, *p* < 0.001]. Also of interest were the differences within a group on day 7: although there was no significant difference between placebo/no-VAP and placebo/VAP sub-groups, there was a highly significant increase in NLR between the probiotic/no-VAP and probiotic/VAP sub-groups [*p* < 0.001]. This result may easily be related to the 3-fold value of lymphocytes [involved as denominator in the neutrophil/lymphocyte formula] compared to placebo sub-groups due to the known issue of immune paralysis.

NLR is one of the most accepted prognostic indices and has a positive correlation to disease severity, progress, and mortality, reflecting the immune system’s sufficiency and its ability to “fight” inflammation. It is thus applicable to a variety of disease conditions, from carcinomas to inflammatory bowel and cardiovascular diseases [48,49,50,51,52,53]. In a recent study of ours on cardiac surgery patients, the NLR values on post-operative days 5 and 7 demonstrated significant discriminatory efficacy for predicting mortality within 90 days [54]. In a meta-analysis, an NLR value greater than 12.65 was found to be associated with bacteremia and was predictive of bacterial sepsis, whereas lower NLR values (cut-off point below 2.06) showed an association with influenza virus infection in patients with lung infections [55]. In the same manner, the NLR index was demonstrated to be the only biomarker able to discriminate between survivors and non-survivors among major trauma patients [56]. Among the 1694 patients retrospectively evaluated, the authors found that patients with a preoperative NLR value greater than 3.23 experienced increased mortality and longer ICU stays [57]. Similarly, Larmann et al. [58] confirmed a preoperative predictive value of NLR greater than 3.1 for patients facing major adverse events. In our study, we found greater values of around 5 on day 0. However, this finding was easily explainable by the inflammatory response elicited by both the major trauma itself and the psychological stress of the motor vehicle crash, leading to the activation of the neuroendocrine system and thus the release of cytokines and stress hormones [59]. We knew this because the blood sample was obtained a few hours after these events.

As a common reaction to an overwhelming inflammatory response, the critically ill patient generally experiences reduced lymphocyte numbers and activity as a result of B- and T-cell apoptosis [60,61]. Besides lymphocytopenia, the increase in the number of neutrophils, as well as “wrong” systemic neutrophil activation and migration towards the microvasculature, also promotes further tissue injury and multiple organ failure [62]. A recent study positively correlates the severity of brain trauma with the number of neutrophils in peripheral blood and brain tissue [63]. Seventy-two critically ill multi-trauma patients were allocated to a 15-day treatment with a formula of four probiotics plus prebiotics [Synbiotic 2000Forte; Medipharm, Sweden, containing *Pediococcus pentosaceus 5-33:3, Leuconostoc mesenteroides 32-77:1*, *L. paracasei* ssp. *paracasei 19*; and *L. plantarum* 2362 at a concentration of 10^11^ cfu each one] or maltodextrin as a placebo. VAP occurred in 5 and 12 patients (13.9% vs. 33.3%, *p* = 0.047] and sepsis in 5 patients and 13 patients (13.9% vs. 36.1%, *p* = 0.028) treated with synbiotics compared to the placebo. White blood cell counts were significantly lower in synbiotics-treated patients on post-trauma days in patients who did or did not develop VAP or sepsis [14]. In an observational study of 68 participants diagnosed with sepsis within the first 24h due to VAP or other nosocomial infections, the absolute count of CD3(+)/CD4(+) lymphocytes was found to be significantly lower (*p* = 0.034), while the apoptosis of isolated monocytes significantly increased (*p* = 0.007), in sepsis patients due to VAP in comparison to other infections [64]. Similar findings were also reported in a group of 40 patients suffering from COVID-19 infection: the flow cytometry results of peripheral blood samples examined for lymphocyte subsets revealed significant and sustained reductions in lymphocytes, but increases in neutrophils, directly associated with disease severity, as also occurred with the NLR index [65].

These alterations in neutrophil and even greater changes in lymphocyte numbers directly mirror a valuable increase in the NLR, which occurs due to a significant increase in the nominator [neutrophils] and also to a decrease in the denominator [lymphocytes], as also seen in our cases. However, in the situation of severe multi-trauma, for individuals receiving probiotics as a part of their intensive care treatment, a differentiation in NLR components—that is neutrophils and lymphocytes—is to be expected, since, in practice, NLR expresses the immune profile of the patient and consequently his/her inflammatory reaction, which is greatly improved by probiotics [66,67,68].

It is also well known that probiotics can potentially suppress various inflammatory states in the intestines by mitigating the inflammatory process, enhancing the mucosal and epithelial barriers, and counteracting colonization by pathogens. However, a lot of these properties are strain-specific since some strains are potent inducers of regulatory cells, while others are not [69,70]. This is only shown in a few studies: in severely ill patients who received synbiotics [*L. casei*, *L. rhamnosus*, *L. acidophilus*, *L. bulgaricus*, *Bifidobacterium breve*, *Bifidobacterium longum*, *Streptococcus thermophilus*, and fructo-oligo-saccharides as prebiotics], a statistically significant decrease in the NLR index [from 7.83 to 6.01, *p* = 0.04] was evident, in parallel with a statistically significant reduction in endotoxin levels and inflammation markers in relation to the placebo the group [71]. There were similar findings in relation to other diseases: *Bifidobacterium* and *Lactobacillus* spp. were shown to ameliorate oxidative stress as well as inflammatory markers and thus significantly improve the NLR index in patients suffering from diversion colitis [72]. A probiotic mixture of *Bifidobacterium longum* Bar33 and *Lactobacillus helveticus* Bar13 improved immune function in the gut and the periphery in elderly adults [27], while the probiotic *Bifidobacterium longum* combined with an extract from the mushroom Lentinula edodes mycelia was found to control the T-regulatory and dendritic cell phenotypes to produce anti-inflammatory responses after azithromycin treatment for 5 days [73]. In a model of acute respiratory distress syndrome, *Lactocaseibacillus rhamnosus* treatment was found to exert an anti-inflammatory effect by decreasing the proinflammatory cytokine-associated Th1 and Th17 responses, thus inhibiting neutrophil infiltration into lung cells and finally reducing the NLR index [74]. In the same model receiving broad-spectrum antibiotics for 8 weeks, peroral treatment with the VSL#3 compound, consisting of *Streptococcus thermophilus*, *Bifidobacterium breve*, *B. longum*, *B. infantis*, *Lactobacillus acidophilus*, *L. plantarum*, *L. paracasei*, and *L. delbrueckii* subsp, produced similar results. *Bulgaricus* exhibited extensive control of mucosal, peripheral, and systemic innate and adaptive immunity [26].

In the present study, a combo regime of the well-studied probiotics *L. acidophilus* LA-5, *L. plantarum* UBLP-40, *B. animalis* subsp. *lactis* BB-12 and *S. boulardii* Unique-28 was used, with these species all having documented anti-inflammatory and immunomodulatory properties [19,20,75,76]. This is why, after the generation of the ROC curve for probiotic-treated patients we found—in addition to the number of patients experiencing VAP being small—that a NLR7 cut-off value of 7.20 was a prognostic factor of VAP (AUC: 78.6%, *p* = 0.015, 95% CI: 62.6–94.5%), and has a high specificity of 90.2% and a sensitivity of 42.9%. The high specificity of this cut-off value is clearly seen in the difference of NLR7 values in the two sub-groups of probiotic-treated patients: those [*n* = 52] showing NLR7 4.18 ± 2.22 did not experience VAP, while those [*n* = 7] with NLR7 8.8 ± 1.11 finally presented VAP, despite probiotic treatment. In other words, in patients treated with probiotics—known for having immune-regulatory and anti-inflammatory properties, and finally helping severely ills patient to not exert lymphopenia—a NLR7 value equal or higher than 7.20 may be considered as an alert or as a specified index for an upcoming VAP.

Although the findings of this post hoc analysis impressively documented the immunological status of each of the 4 sub-groups of the ProVAP trial, and underlined the beneficial defense effects exerted by these probiotics, there were some limitations. Firstly, because it was a post hoc analysis and not primary research, the sample size estimation was based on the principal outcomes of the ProVAP study. The absence of power analysis was the reason for the limited VAP events in the group of probiotic-treated patients—our precise analysis related only to information obtained from the ProVap trial versus the 15 cases in the placebo. Conversely, this significant difference reflected the beneficial effects of probiotic use. The second point was the arbitrary decision to compare white blood cell counts on day 0 with those of day 7; this was mainly based on the post-trauma day on which VAP first manifested, although this decision may have contributed to interventional bias. And the third point, directly related to the first, was that our analysis did not allow for conclusive results to be drawn, but only the recognition of significant differences and a clear cut-off value as a prognostic factor of VAP for patients treated with probiotics only. We hope that this clear cut-off value offers a valuable literature background which explains the findings.

## 5. Conclusions

In conclusion, the index derived from the white blood cell count, namely the NLR, may be considered a plausible prognostic biomarker in critically ill patients, since it can enable the evaluation of the defense status of the patients’ immune system and the possibilities/likelihood of resistance or recovery after immunomodulatory enhancement by means of well-selected probiotic strains. Although the results of the present post hoc investigation should be assessed with caution due to the small number of patients experiencing VAP in the probiotics group, the NLR7 cut-off value of 7.2 found in this group seems having a high specificity of 90.2% as a prognostic factor of VAP. However, in order to speak of a cut-off value in such cases, there needs to be a significant amount of data from prospective studies before safe causative conclusions can be drawn, and the conclusions may finally prove to be essentially species- or strain-specific. Nevertheless, in this era of personalized medicine, the NLR index is of predictive value in treatment decisions concerning the critically ill.

## Figures and Tables

**Table 1:** Neutrophils counts on days 0 and 7.

Groups	Day 0	Day 7	*p* Value *
Placebo (*n* = 53)	6.94 ± 2.28	12.68 ± 5.43	<0.001
Probiotics (*n* = 59)	6.21 ± 2.00	11.68 ± 5.05	<0.001
*p* Value **	0.07	0.2	

* *p* value after paired *t*-test application; ** *p* value after *t*-test for independent samples.

**Table 2:** Neutrophils in no-VAP and VAP sub-groups, on days 0 and 7.

Groups	Sub-Groups	Day 0	Day 7	*p* Value *
Placebo (*n* = 53)	no-VAP (*n* = 38)	7.20 ± 2.22	13.08 ± 6.16	<0.001
	VAP (*n* = 15)	6.31 ± 2.37	11.70 ± 6.14	0.012
*p* Value **		0.2	0.4	
Probiotics (*n* = 59)	no-VAP (*n* = 52)	6.28 ± 1.96	11.31 ± 5.09	<0.001
	VAP (*n* = 7)	5.71 ± 2.38	14.32 ± 4.09	<0.001
*p* Value **		0.02	0.14	

* *p* value after paired *t*-test application; ** *p* value after *t*-test for independent samples.

**Table 3:** Lymphocytes counts on days 0 and 7.

Groups	Day 0	Day 7	*p* Value *
Placebo (*n* = 53)	1.30 ± 0.37	0.86 ± 0.43	<0.001
Probiotics (*n* = 59)	1.23 ± 0.29	2.95 ± 1.23	<0.001
*p* Value **	0.3	<0.001	

* *p* value after paired *t*-test application; ** *p* value after *t*-test for independent samples.

**Table 4:** Lymphocytes in no-VAP and VAP sub-groups, on days 0 and 7.

Groups	Sub-Groups	Day 0	Day 7	*p* Value *
Placebo (*n* = 53)	no-VAP (*n* = 38)	1.33 ± 0.36	0.88 ± 0.41	<0.001
	VAP (*n* = 15)	1.22 ± 0.41	0.81 ± 0.50	0.06
*p* Value **		0.3	0.6	
Probiotics (*n* = 59)	no-VAP (*n* = 52)	1.24 ± 0.29	3.02 ± 1.21	<0.001
	VAP (*n* = 7)	1.16 ± 0.33	2.41 ± 1.28	0.03
*p* Value **		0.5	0.2	

* *p* value after paired *t*-test application; ** *p* value after *t*-test for independent samples.

**Table 5:** Neutrophils to Lymphocytes ratio on days 0 and 7.

Groups	Day 0	Day 7	*p* Value *
Placebo (*n* = 53)	5.61 ± 2.06	16.15 ± 5.81	<0.001
Probiotics (*n* = 59)	5.18 ± 1.62	4.71 ± 3.44	0.066
*p* Value **	0.2	<0.001	

* *p* value after paired *t*-test application; ** *p* value after *t*-test for independent samples.

**Table 6:** Neutrophil-to-lymphocyte ratio in no-VAP and VAP sub-groups on days 0 and 7.

Groups	Sub-Groups	Day 0	Day 7	*p* Value *
Placebo (*n* = 53)	no-VAP (*n* = 38)	5.74 ± 1.65	16.43 ± 6.07	<0.001
	VAP (*n* = 15)	5.29 ± 1.65	15.45 ± 5.19	0.001
*p* Value **		0.4	0.3	
Probiotics (*n* = 59)	no-VAP (*n* = 52)	5.19 ± 1.61	4.18 ± 2.22	0.01
	VAP (*n* = 7)	5.12 ± 1.77	8.8 ± 1.11	0.018
*p* Value **		0.98	<0.001	

* *p* value after paired *t*-test application; ** *p* value after *t*-test for independent samples.

## Data Availability

The data that support the findings of this study are available on request from the corresponding author, [K.K.].
